# Draft Genome Sequences of Five Novel Polyketide Synthetase-Containing Mouse *Escherichia coli* Strains

**DOI:** 10.1128/genomeA.01082-16

**Published:** 2016-10-06

**Authors:** Anthony Mannion, Zeli Shen, Yan Feng, Alexis Garcia, James G. Fox

**Affiliations:** Division of Comparative Medicine, Massachusetts Institute of Technology, Cambridge, Massachusetts, USA

## Abstract

We report herein the draft genomes of five novel *Escherichia coli* strains isolated from surveillance and experimental mice housed at MIT and the Whitehead Institute and describe their genomic characteristics in context with the polyketide synthetase (PKS)-containing pathogenic *E. coli* strains NC101, IHE3034, and A192PP.

## GENOME ANNOUNCEMENT

*Escherichia coli* is typically considered a harmless commensal bacteria colonizing the mammalian intestine. However, infection by numerous strains from the B2 phylogroup can cause septicemia/bacteremia, urinary tract infection, and meningitis and are increasingly being linked to inflammatory bowel disease and colorectal tumors in humans (1–3). These strains commonly harbor an ~54-kb polyketide synthetase (PKS) pathogenicity island that produces a genotoxin called colibactin (4, 5). Recently, colibactin expression by the mouse commensal *E. coli* strain NC101 was shown to potentiate colitis-associated colorectal cancer in IL-10^−/−^ mice (2). Furthermore, aside from causing double-strand DNA breaks, colibactin facilitates intestinal colonization and systemic translocation that causes septicemia and meningitis (6). Thus, the virulence and oncogenic modalities of colibactin-producing *E. coli* strains in experimental mice may represent a potential confounder of results, especially in models of inflammatory disease and cancer.

In this report, we announce the draft genomes of five novel B2-phylotype *E. coli* strains isolated from the feces of two asymptomatic surveillance mice, the uterine fluid of a transgenic mouse, and from the blood of two experimental immunocompromised mice with sepsis housed under specific-pathogen-free conditions. We previously found all these strains except A4 were PCR-positive for the PKS genes clbA and clbQ and caused megalocytosis and γ-H2AX-detectable DNA damage in HeLa cells *in vitro*, suggesting colibactin activity by these organisms ([7]; our unpublished data). We briefly describe the features of these novel *E. coli* genomes in context to the previously sequenced genomes of NC101 and the PKS-containing septicemia- and meningitis-causing strains IHE3034 and A192PP (4, 6).

Genomes were sequenced using PacBio RSII and resulting sequencing data were assembled into two to five contigs using RS_HGAP_Assembly.3 from the SMRT Portal 2.3. Contigs were annotated using the RAST tool kit (RASTtk) (8). Novel strains have genomic sizes, G+C content, protein-coding sequences, and RNAs comparable to those of the genomes of NC101 (GenBank: NZ_AEFA00000000.1), IHE3034 (GenBank: NC_017628.1), and A192PP (GenBank: NZ_CVOH00000000.1) (Table 1). All novel genomes except A4 contained complete PKS islands, with all genes having ≥99% sequence identity and coverage as well as identical synteny to those from NC101, IHE3034, and A192PP.

**TABLE 1:** Genomic characteristics of novel mouse *E. coli* strains

Strain	GenBank accession no.	Origin of mouse isolates	Genome size (bp)	No. of contigs	Fold coverage	G+C content (%)	No. of protein-coding sequences	No. of RNA sequences
*E. coli* 1409150006 (MIT A2)	MBNU00000000	Feces	5,292,961	3	114.7	50.5	5,203	108
*E. coli* 1409160003 (MIT A4)	MBNV00000000	Feces	5,066,411	4	166.4	50.5	5,003	114
*E. coli* 1408270010 (MIT A21)	MBNW00000000	Uterine fluid	5,318,023	5	129.9	50.5	5,280	108
*E. coli* 1512290008 (Whitehead Institute 0008)	MBNX00000000	Blood	5,289,663	3	147.7	50.5	5,203	108
*E. coli* 1512290026 (Whitehead Institute 0026)	MBNY00000000	Blood	5,005,444	2	140.6	50.6	4,876	108

PATRIC’s proteome comparison service was used to determine the number of homologous genes between NC101, IHE3034, and A192PP as references versus the novel *E. coli* strains (9). NC101 and the novel PKS-containing strains shared >4,780 proteins (>97%) as homologs, whereas between NC101 and A4, 4,732 proteins (~89%) were homologous. All novel *E. coli* strain shared considerably less homologs to IHE3034 (4,386 to 4,525 proteins; ~87 to 90%) and A192PP (4,472 to 4,624; ~80 to 83%).

Aside from PKS, the virulence factors enterobactin siderophore receptor protein, s-fimbriae minor subunit, glutamate decarboxylase, per-activated serine protease autotransporter enterotoxin, and bor protein precursor were identified in NC101 and the novel PKS-containing strains using VirulenceFinder 1.5 (10). A4 also contained glutamate decarboxylase, per-activated serine protease autotransporter enterotoxin, and bor protein precursor as well as serine protease autotransporters of *Enterobacteriaceae*.

Together, these genomic data suggest that the novel PKS-containing strains are more similar to NC101 than IHE3034 and A192PP. Future studies will investigate the pathogenic and carcinogenic potential of these and other PKS-containing *E. coli* strains in mice under differing experimental protocols.

### Accession number(s).

The genome sequences have been submitted to GenBank under the accession numbers listed in Table 1.
